# Editorial: Progress in understanding the immunogenetic basis of disease susceptibility and outcomes

**DOI:** 10.3389/fgene.2022.1007825

**Published:** 2022-08-25

**Authors:** Tina Bharani, Divyansh Agarwal

**Affiliations:** ^1^ Department of Surgery, Thomas Jefferson University Hospital, Philadelphia, PA, United States; ^2^ Department of Surgery, Brigham and Women’s Hospital, Boston, MA, United States; ^3^ Department of Biology, Massachusetts Institute of Technology, Cambridge, MA, United States

**Keywords:** Immunogenetics, HLA, haplotype, natural killer cells, preeclaimpsia, pregnancy

Advances in sequencing technologies over the last 2 decades have dramatically increased our understanding of the molecular and genetic basis of immune responses, which in turn has led to a better understanding of the mechanisms underlying immune tolerance and surveillance. Similarly, better human leukocyte antigen (HLA) typing has helped explain how different genomic signatures might affect the development of autoimmune diseases, and opened new avenues of investigation in transplantation. The aim of our research topic was to collect articles that present the most cutting-edge, relevant, and impactful pieces on the role and study of immunogenetic polymorphisms affecting differential susceptibility to human diseases.

The research topic was open between 4 June 2021 to 21 March 2022. Four manuscripts were accepted for publication. These articles have increased our understanding of the role of immunogenetics in the heterogeneous response to COVID-19 infection, helped us appreciate the ethnic differences in genetic make-up of autoimmune diseases such as type 1 diabetes, acknowledged the high frequency of homozygosity that exists in Saudi Arabia population, and reviewed the role of HLA in causing preeclampsia in pregnant women.

The study performed by Maruthamuthu et al. demonstrated the role of HLA class I in regulation of NK cells and response to severe acute respiratory syndrome coronavirus-2 (SARS-CoV-2) infection. Highly polymorphic killer cell immunoglobulin-like receptors (KIR) and the corresponding HLA class I molecules regulate NK cell maturation and function, which in turn determines the severity of COVID-19 infection. Patients hospitalized for COVID-19 infection had lower frequency of KIR3DL1+HLA-Bw4+ and KIR3DL2+HLA-A3/11+ combinations, which were responsible for inadequate NK cell maturation. On the contrary, an increased frequency of KIR2DS1+KIR2DS5+ in severe COVID-19 patients suggests vigorous NK cell response triggered via these activating receptors, and subsequent production of exuberant inflammatory cytokines contributing to severe COVID-19. This research study highlighted the immunogenetic variables, primarily differential combinations of KIR-HLA, that regulate the dual role of NK cells as anti-inflammatory and pro-inflammatory mediators.

A prospective study by Al Yafei et al. highlighted the differences that exist in HLA class II alleles and haplotypes associated with Type 1 diabetes mellitus (T1D) among various ethnicities. The study enrolled 149 subjects with T1D and 147 subjects with normoglycemia and sequenced their HLA class II genotypes. Most common DRB1 and DQB1 haplotypes and alleles associated with T1D in Emirati population were similar to the ones identified with other Middle Eastern, Caucasian and non-Caucasian populations, while showed no similarity to HLA class II alleles identified in European, Asian and African patients with T1D.

Saudi Arabia has one of the highest rates of consanguinity in the world at 57.7%. The retrospective study by Chentoufi et al. showed very low diversity of HLA class I and II alleles and haplotypes in Saudi population. Only 15 HLA-A, 20 HLA-B, 11 HLA-C, 13 HLA-DRB1, and five HLA-DQB1 homozygous alleles were identified in 2,773 subjects who were admitted at a tertiary hospital between 2012 and 2016. The highest homozygosity in HLA class I was in locus C, followed by locus A then B and the highest homozygosity in HLA class II was in DQB1 compared to DRB1. This high frequency of homozygosity documented in the Saudi population has the potential to further guide practices in transplantation, strategies to prevent transfusion-related graft versus host disease, and management of recessive mutation expressions in this population.

The article by Aisagbonhi and Morris reviewed the role of classical and non-classical HLA during pregnancy and pre-eclampsia. Given that the fetus is semi-allogeneic, maternal immunologic tolerance to the fetus is developed through interaction between fetal trophoblast cells and maternal immune cells at the placental interface. The proposed mechanisms for development of preeclampsia include maternal factors unrelated to placenta, maternal-fetal incompatibility and deficient spiral artery remodeling. The maternal factors that have been associated with increased risk of preeclampsia include obesity, cardiovascular disease, autoimmune diseases including SLE and rheumatoid arthritis, which manifest HLA class II and HLA-B alleles. None of HLA molecules are expressed at the placental interface. Thus, further work is needed to identify the HLA alleles at placental interface that may play a role in maternal factors causing preeclampsia. The two most common HLA molecules that have been identified to be dysregulated in preeclampsia include HLA-A and HLA-G. Decreased levels of circulating and placental HLA-G have been identified in pre-eclampsia, leading to increased expression of pro-inflammatory cytokines, decreased T regulatory cells and defective spiral artery remodeling. In some studies, inhibitory KIR genotypes have been associated with preeclampsia, whereby interactions between HLA-C and KIR on NK cells have failed to stimulate pro-migratory chemokines and cytokines, thus leading to defective spiral artery remodeling.

Greater understanding of immunogenetics will help appreciate the physiologic and pathophysiologic nature of immune defenses, and help predict the response of immune cells to environmental stressors. These insights can further advance precision immunology as a field. Altogether, the papers published as a part of this collection demonstrate that unraveling the genetic basis of immune response as well as the identification of genetic variations that result in immune defects are fundamental to the discovery of new therapeutic targets for immune-mediated diseases.

